# Diffusion-Weighted Images and Contrast-Enhanced MRI in the Diagnosis of Different Stages of Multiple Sclerosis of the Central Nervous System

**DOI:** 10.7759/cureus.41650

**Published:** 2023-07-10

**Authors:** Mashael A Ismail, Naglaa M Elsayed

**Affiliations:** 1 Radiologic Sciences, Faculty of Applied Medical Sciences, King Abdullah Medical Complex, Ministry of Health, Jeddah, SAU; 2 Radiologic Sciences, Faculty of Applied Medical Sciences, King Abdulaziz University, Jeddah, SAU; 3 Diagnostic Radiology, Faculty of Medicine, Cairo University, Cairo, EGY

**Keywords:** delayed enhancement, enhancement time, diffusion weighted images, magnetic resonance imaging, multiple sclerosis

## Abstract

Introduction

Multiple sclerosis (MS) is one of the most prevalent disorders of the central nervous system (CNS), and it can be observed in the field of radiological cross-sectional magnetic resonance imaging (MRI). The prevalence of MS in Saudi Arabia has increased as compared to the past few years. MRI is the gold standard non-invasive modality of choice in MS diagnosis according to the National Multiple Sclerosis Society (NMSS), New York City. This study aimed to highlight the significance of using diffusion-weighted images (DWIs) and the use of contrast media in the MS protocol, as well as the importance of identifying the suitable time of imaging after contrast enhancement to detect active lesions.

Methods

A retrospective cross-sectional study was conducted of 100 MS patients with an age range of 17 to 56 years. The data set included 41 active cases and 59 inactive cases. All patients had an MRI standard protocol of both the brain and spine in addition to DWI sequence and contrast agent (CA) injection, with images taken in early and delayed time.

Results

Of the patients, 71% were female and 29% were male. Active MS disease was more significant at younger ages than at older ages. Active lesions were significantly enhanced in delayed contrast images and showed high signal intensity in both the DWI and apparent diffusion coefficient (ADC) map, while inactive lesions showed no enhancement after contrast injection and showed an iso-signal intensity in both the DWI and ADC map.

Conclusion

The use of CA has developed over the years in the diagnosis of MS patients. In this study, the relationship between active lesions, DWI, and delayed contrast enhancement is very strong. In future research, we recommend adding a DWI sequence for the suspected active MS spine lesions in addition to delayed enhancement time in active MS after contrast injection to increase MRI sensitivity toward active MS lesions of the brain and spinal cord as well.

## Introduction

Multiple sclerosis (MS) is one of the most prevalent disorders of the central nervous system (CNS), and it can be observed in the field of radiological cross-sectional magnetic resonance imaging (MRI) [1,2]. The World Health Organization (WHO) and the Multiple Sclerosis International Federation (MSIF) worked in cooperation to produce the first Atlas of MS, which was published in 2008 [3]. In 2020, according to the MSIF, 87% of MS patients participated worldwide, in around 115 countries [4].

The Kingdom of Saudi Arabia (KSA) started its first multicenter, national MS registry in 2015. Currently, 20 hospitals from multiple regions of the KSA are listed in the registry. At the national level, the prevalence of MS in Saudi Arabia in 2018 was estimated to be 40.40/100,000 societies overall and 61.95/100,000 Saudi nationals [5].

Usually, the first presentation of MS symptoms is dysesthesia, which causes a squeezing sensation around the torso and makes the patient feel like a blood pressure cuff is tightening [6]. The most prevalent symptom in MS patients is fatigue, with occurrence rates ranging from 53 to 90% [7]. The second most common symptom is numbness or tingling, which might be mild or severe [8]. Spasticity can negatively impact mobility and balance and be a factor in the high level of disability among MS patients [9]. Another common symptom is walking difficulties including issues with balance (ataxia) or weakness in the leg muscles including the sensory deficit from the numbness [10]. When it comes to vision complications, MS may cause optic neuritis, including dim and/or blurred vision or loss of color vision, which can also lead to diplopia (double vision) and nystagmus [11]. Vertigo affects 7-30% of MS patients; it is another disorder linked to the damaged area with lesions in the complex neural structures that connect visual, spatial, and other inputs to the brain that are necessary for producing and maintaining balance [12]. Many other symptoms, such as bladder dysfunction, sexual dysfunction, and depression, have been studied in a cohort study, and the researchers noticed an increase in the symptoms during the years of MS development [13]. The consequences of these limitations on MS patients affect their quality of life and their capability to carry out routine duties.

The standard definition of an acute inflammatory MS relapse is a new or worsening neurological disorder lasting more than 24 hours without a fever or infection linked to one or more of the symptoms listed above [14].

The presence of neurological symptoms and signs after white matter lesions is essential for the main clinical diagnosis of MS. Diagnosing MS lesions involves an MRI scan of the brain and spinal cord with the use of a contrast agent (CA) to identify the active lesions, cerebrospinal fluid (CSF) analysis, and laboratory tests. The use of McDonald’s criteria makes it simpler to distinguish between MS and other neurological disorders. Disseminated in time (DIT) and disseminated in space (DIS) are the main concepts of these criteria [15,16]. To define DIS, lesions must affect at least two areas of the CNS to be considered. The development of CNS lesions over time refers to the DIT [17].

The McDonald criteria for diagnosing MS patients depend on the following descriptions [18]:

• Having two or more attacks and two or more objectively observable lesions clinically.

• Having two or more attacks with one objectively observable lesion clinically and a clinical history that suggests a prior lesion.

• Having two or more attacks with one objectively observable lesion clinically and one of these criteria: DIS in an additional attack or an MRI showing DIS in two or more areas of the CNS lesions.

• Having one clinical attack, the clinically isolated syndrome (CIS) with two or more objectively observable lesions clinically and one of these criteria: DIT in an additional attack or an MRI showing DIT for both enhancing and non-enhancing lesions or by having new hyperintense lesions in a new MRI scan compared with a previous one or by the detection of CSF-specific oligoclonal bands.

• CIS with one objectively observable lesion clinically and one of these criteria: DIS and/or DIT in an additional attack or an MRI showing DIS in two or more areas of the CNS lesions or MRI showing DIT for both enhancing and non-enhancing lesions or by having new hyperintense lesions in a new MRI scan compared with the previous one or the detection of CSF-specific oligoclonal bands.

• Not all MS patients have attacks; some have primary progressive multiple sclerosis (PPMS). PPMS has been described as a decline in neurological function since the disease was first identified. The diagnostic criteria are at least one year of disease progression clinically and either one or more brain T2 lesions, two or more spin lesions, or the detection of CSF-specific oligoclonal bands.

Aims

The aim of this study was to study the diagnostic abilities of diffusion-weighted images (DWIs) and contrast-enhanced (CE) MRI of stationary and active MS lesions of the brain and spine. This study aimed to highlight the significance of using DWI and the use of contrast media (CM) in the MS protocol and the importance of identifying the suitable time of imaging after contrast enhancement to detect active lesions.

Background

MS is one of the most commonly diagnosed inflammatory autoimmune disorders of the CNS. MS is generated by genetic and/or hormonal effects [19]. Another cause of MS is neurological damage after trauma in adolescents. [20]. Additionally, certain environmental factors such as obesity, smoking, and mononucleosis contribute to and raise the possibility of the occurrence of this disease. Another environmental effect corresponds to seasonal variations in sunshine exposure influencing vitamin D levels, which are more common in countries with more temperate climates. MS is interspersed with totally or partially reversible neurological impairment episodes that typically last days to weeks. MS includes different symptoms, such as vision loss as a result of optic neuritis, limb weakness, sensory loss, and double vision [21]. MS affects about 2.8 million people worldwide [4]. Women are more likely than men to have MS, and this incidence has increased over the past few years [22,23]. MS primarily affects adolescents, most often manifesting between 20 and 40 years of age, but it can also develop in children before the age of 18 [24].

MRI is the radiological modality of choice for diagnosing and following up on MS progression. MRI has the potential to evaluate MS treatment efficacy [25]. MRI assists in the detection of MS lesions with the use of McDonald’s criteria, depending on the time and location [20]. McDonald’s criteria is a method using radiographic, clinical, and laboratory diagnostic tests to diagnose MS disease, and it was first presented in 2001 [18,26]. The dissemination of MS lesions suggests disease activity and points to the need for changing therapies [27]. The baseline MRI protocol for brain MS mainly uses sagittal T2 fluid attenuation inversion recovery (FLAIR), and axial T2 and T1 fast spin echo (FSE), and adds a post-contrast single dose of 0.1 mmol/kg axial T1-weighted spin echo [25]. Each sequence has its own utility in identifying lesions; T2-weighted imaging (T2-WI) can detect chronic MS lesions [28,29]. Lesions located in the periventricular area are difficult to visualize in T2-WI due to the high signal intensity from the surrounding CSF. The best method to see periventricular lesions is by using T2-FLAIR sequence axial and sagittal orientations [30]. T2-FLAIR sequences are heavily T2-WI and null the fluid, increasing the background intensity of lesions in the white matter region [31]. One of the main issues in imaging MS brain lesions in MRI is the appearance of active lesions. Sometimes, it is obvious from the clinical diagnosis, but by the radiologic way, it cannot be diagnosed well [32]. MS lesions in diagnostic images cannot be clearly seen due to many factors, including time management in the imaging protocols after CA administration [33].

DWIs describe the movement of water molecules. The apparent diffusion coefficient (ADC) is a quantitative measurement combined with DWI detected in MS during myelin breakdown. The alteration of the inflammatory process results in changes to DWI and ADC to be observed [34]. Nowadays, DWI is routinely fixed in MS MRI protocols. This purpose is used to identify active lesions with the restriction of diffusion [35]. On some occasions, acute cases can be diagnosed if there is an increase in the intensity of DWI with post-contrast T1-weighted imaging (T1-WI) in MRI scans [36,37].

One of the main characteristics of MS is the breakdown of the blood-brain barrier (BBB). Active inflammation causes BBB damage, which is detected by T1-WI sequences with a CA [38]. CA injection helps differentiate between active and inactive MS lesions. CA identifies areas of MS lesions, and the degree of enhancement acts as an indicator of the quantity of current inflammation [39]. Different split doses of CM injection are valuable in some cases of MS. The use of a dose of gadolinium in MS patients at a 5-minute interval improved the ability to identify active lesions [40]. For DIT in MS patients, the use of gadolinium-based contrast agents (GBCAs) is crucial [41]. The enhanced lesions in follow-up MRI scans provide information about the DIT [42].

According to McDonald’s 2017 diagnostic criteria, concerns about the importance of monitoring the diagnostic and safety of intravenous (IV) gadolinium CA have been raised. Standardized imaging protocols were approved as guidelines by the 2015 Magnetic Resonance Imaging in Multiple Sclerosis (MAGNIMS) and the 2016 Consortium of Multiple Sclerosis Centers (CMSC) on the use of MS MRI diagnosis [25,41]. MAGNIMS is a group of networks consisting of academics from around Europe who are interested in using MRI to study MS [30]. CMSC is the leading group of healthcare professionals dedicated to enhancing the lives of individuals diagnosed with MS [43]. These developments indicate a potential shift in the use of the MS MRI protocol. The suggested protocol in brain MS is ideally performed in 3 Tesla (3 T), axial proton density (PD) or T2-WI, sagittal T2-FLAIR, and post-CM axial T1-WI, as per MAGNIMS. For CMSC, the brain protocol is a 3D inversion recovery (IR), T1 gradient echo (GRE), sagittal T2-FLAIR, axial DWI, and 3D-fast low-angle shot post-CA [44]. A time gap between the CA injection and T1-WI is highly recommended, with a minimum of 5 minutes for follow-up MS MRI scans to check DIT and DIS [25,41]. Spine imaging requires two or three sagittal views, PD-WI, short inversion recovery, T2 images, and phase-sensitive inversion recovery [45]. The use of axial views in spine images is not mandatory, as per MAGNIMS and CMSC.

## Materials and methods

A retrospective cross-sectional study was performed in 2023. The data were collected from King Abdullah Medical Complex Hospital using MRI brain and spine studies performed in the last five years (2019-2023) with the permission of the committee, institutional review board, and Ministry of Health, who gave ethical approval on February 27, 2023, under approval number A01579. The data were collected within two months, from March to April 2023.

This research included 100 patients; 71 were females and 29 were males. Active MS disease comprised 41 cases (27 females and 14 males), ranging in age from 17 to 56 years, with a mean age of 32 ± 8 years. Stationary MS disease included 59 cases (44 females and 15 males), ranging in age from 18 to 57 years, with a mean age of 36 ± 9 years.

The inclusion criteria ensured that patients clinically diagnosed with MS disease and identified the activity type also had an MRI of the brain and spine. The stationary and active cases were classified based on the clinical diagnosis. The exclusion criteria included post-operative cases, brain trauma, motion artifacts, and any MS patient who did not have an MRI of the whole spine.

General Electric (GE) and Siemens MRI, 1.5 and 3 T, were used for scanning MS patients with IV CM with both early and delayed enhancement in conjunction with DWI. The immediate image was taken using an MRI after the CA injection was considered the early time. The delayed enhancement time in this research was performed with a minimum of 10 minutes. Gadolinium was the CM used in the current study, with an amount of 0.2 mL per kilogram.

Before administering CM, the patient had to undergo a recent renal function test. The patient was lying down in a supine position, headfirst, and arms beside the body. MRI sequences of the brain included the following:

• Axial FSE T1-WI

• Axial FSE T2-WI

• Axial DWI with ADC map (ADC automatically generated).

• Axial GRE echo

• Sagittal and axial T2-FLAIR

• Post-CM, 3D multiplanar T1-MPRAGE (magnetization-prepared rapid acquisition gradient echo) early enhancement time

• Post-CM, axial FSE T1-WI delayed enhancement time

MRI sequences of the whole spine included the following:

• Axial and sagittal FSE T2-WI

• Post-CM, axial, and sagittal FSE T1-WI early/delayed enhancement time.

All images post-CM injection were obtained using the fat suppression technique.

Data were collected from the PACS system of the hospital and stored securely in a confidential way without any personal information on the patient.

The reading and activity classification depended on the patient’s report, which had already been diagnosed by a consultant neuroradiologist. The signal intensity of the various sequences in the MRI images was used to fill in the Excel sheet from the written report for each of T2-FLAIR, DWI, ADC map, post-contrast T1-MPRAGE for the brain images, and FSE-T2-WI with post-contrast FSE-T1-WI for the spine images. The disease duration, clinical picture, age, and gender were also recorded.

Statistical analysis

The software used in the current study was SPSS Version 25 (IBM Corp., Armonk, NY). The age of the studied patients was presented as the mean and standard deviation (SD). The comparison of age between the active and inactive cases was conducted using a t-student test.

Categorical variables were presented by number and percentage. All categorical data were compared between the active and inactive cases using the chi-square test. In all tests, the p-value was considered significant when it was less than 0.05.

## Results

The research sample included 100 patients, 71% females and 29% males, with a mean age of 34 ± 9 years. The youngest was 17 years old, and the oldest was 57 years old. The results showed 41 active MS patients, with 65.9% females and 34.1% males. Of the patients, 59 did not have active MS disease, with 74.6% females and 25.4% males (Table 1).

**Table 1:** Age and gender in relation to disease activity. MRI, magnetic resonance imaging; F, female; M, male; SD, standard deviation; n, number of MS patients

			MRI diagnosis groups		Total (n=100)	P-value
			Active (n=41)	Not active (n=59)		
Gender	F	Count	27	44	71	0.344
		%	65.9%	74.6%	71.0%	
	M	Count	14	15	29	
		%	34.1%	25.4%	29.0%	
Age	Years	Mean ± SD	32 ± 8	36 ± 9	34 ± 9	0.04

**Figure 1:**
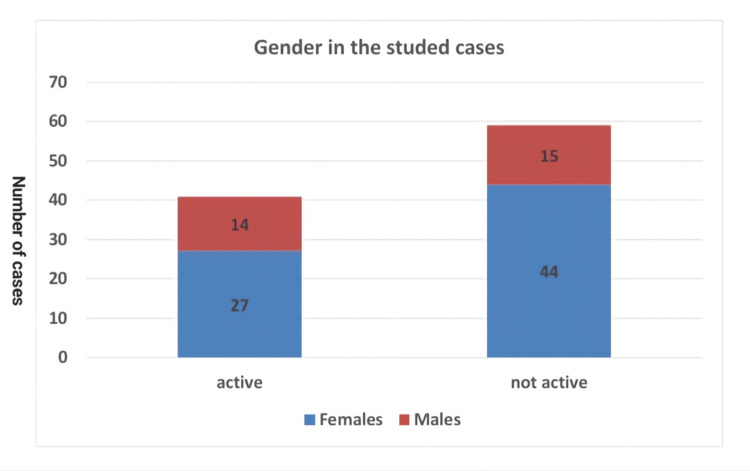
Gender in relation to activity in the studied cases.

**Figure 2:**
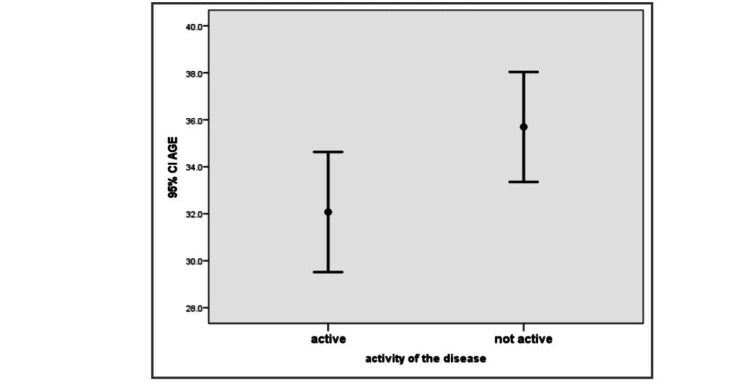
Age in relation to activity in the studied cases. Younger patients showed more activity (P-value < 0.04). CI, confidence interval

The location of MS lesions was classified into three groups: brain only, spine only, and both brain and spine. Of the patients, 20 were active in the brain, eight were active in the spine, and 13 were active in both the brain and spine. For the activity, the results showed no significant difference between gender and activity, but compared to the age groups, active MS disease was more significant in younger individuals than in elderly individuals compared with non-active cases (P-value < 0.04; Figures 1, 2).

Brain findings

**Figure 3:**
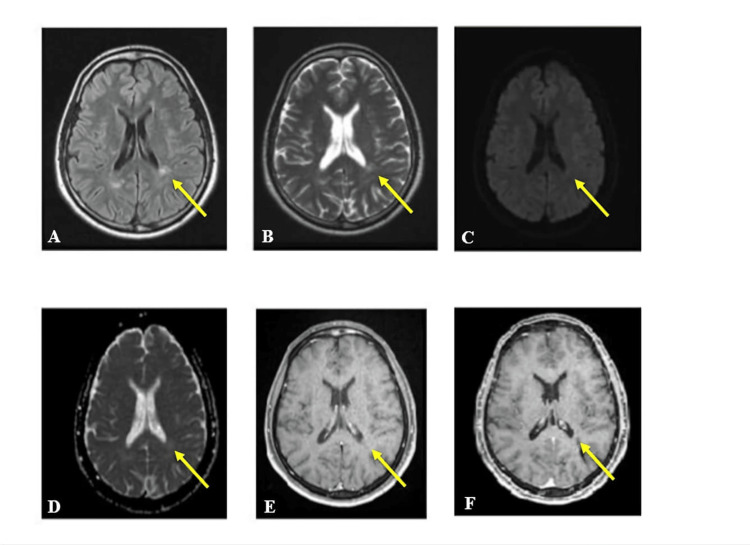
A 30-year-old male patient with inactive MS and subcortical and periventricular white matter lesions. (A) Axial FLAIR showed high signal intensity lesion (yellow arrow). (B) Axial T2 FSE high signal intensity for the same lesion. (C) DWI of the lesion was isointense. (D) ADC map of the same lesion was isointense. (E) Axial T1 FSE post-CE. (F) Axial MPRAGE delayed enhancement time. No enhancement for the lesion at both times. MS, multiple sclerosis; FLAIR, fluid attenuation inversion recovery; FSE, fast spin echo; DWI, diffusion-weighted image; ADC, apparent diffusion coefficient; CE, contrast enhancement; MPRAGE, magnetization-prepared rapid acquisition gradient echo

**Figure 4:**
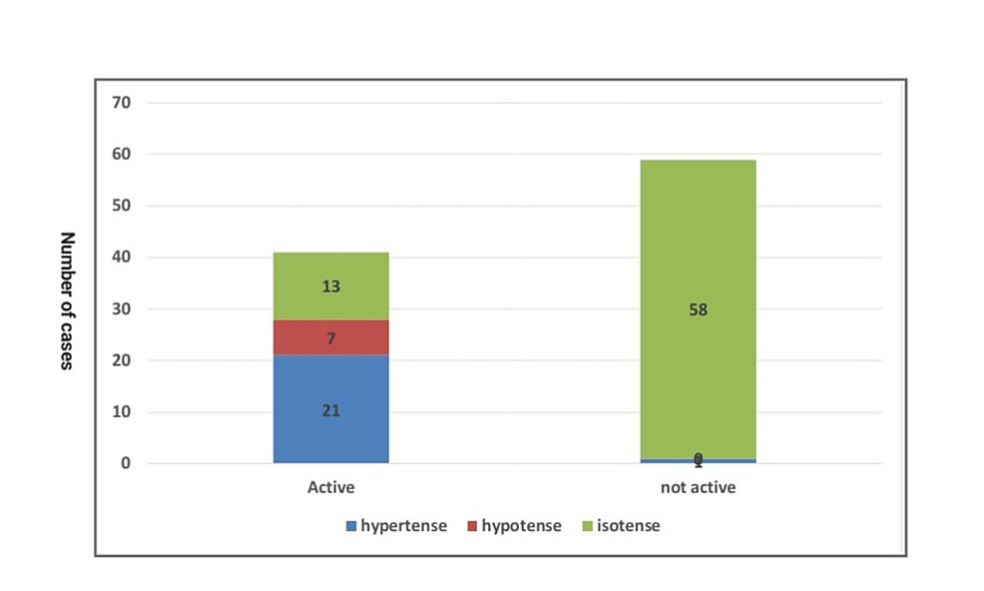
ADC intensity in relation to activity in the studied cases. ADC, apparent diffusion coefficient

Inactive brain cases did not show any enhancement in either early or delayed imaging time for the lesions and showed an iso-signal intensity in either DWI or the ADC map (Figure 3). Inactive lesions were more isointense for DWI and the ADC map than in the active group, with a P-value of <0.01 (Table 2 and Figure 4).

**Table 2:** DWI and ADC in the studied cases. MRI, magnetic resonance imaging; DWI, diffusion-weighted images; ADC, apparent diffusion coefficient; n, number of MS patients

			MRI diagnosis groups		Total (n=100)	
			active (n=41)	not active (n=59)		P-value
Hyperintensity in DWI	No	Count	13	58	71	<0.01
		%	31.7%	98.3%	71.0%	
	Yes	Count	28	1	29	
		%	68.3%	1.7%	29.0%	
ADC intensity	Hypertense	Count	21	1	22	<0.01
		%	51.2%	1.7%	22.0%	
	Hypotense	Count	7	0	7	
		%	17.1%	0.0%	7.0%	
	Isotense	Count	13	58	71	
		%	31.7%	98.3%	71.0%	

**Figure 5:**
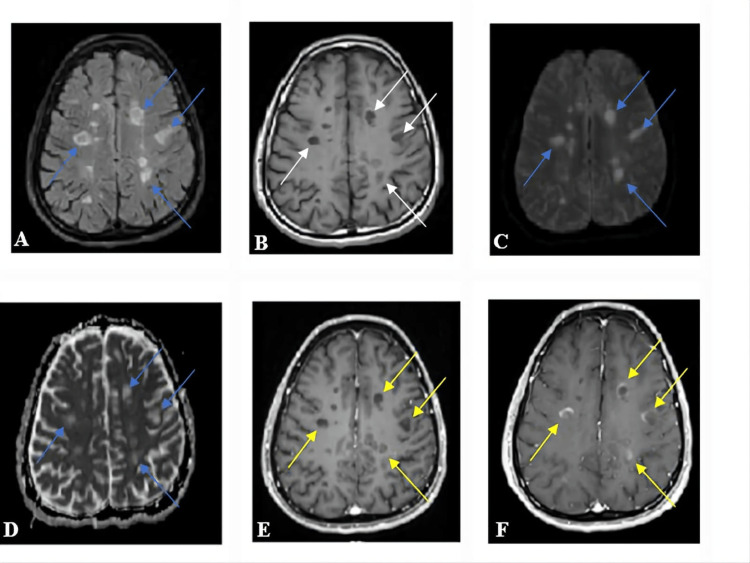
A 38-year-old male patient with active MS and brain periventricular multiple demyelinating plaques. (A) Axial FLAIR showed high signal intensity (blue arrows). (B) Same lesions in axial T1-weighted images were hypointense (white arrows). (C) Axial DWI and (D) the ADC map were hyperintense (blue arrows). (E) Axial MPRAGE in the early post-contrast enhancement with no contrast uptake in any of the lesions (yellow arrows). (F) Delayed phase enhancement; the lesions were fully enhanced (yellow arrows). MS, multiple sclerosis; FLAIR, fluid attenuation inversion recovery; DWI, diffusion-weighted image; ADC, apparent diffusion coefficient; MPRAGE, magnetization-prepared rapid acquisition gradient echo

**Figure 6:**
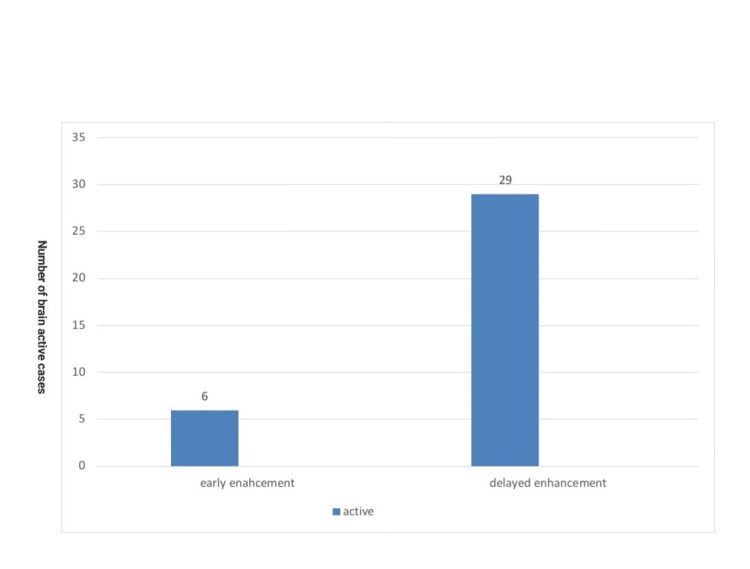
Early and delayed enhancement in the brain in the studied cases.

**Figure 7:**
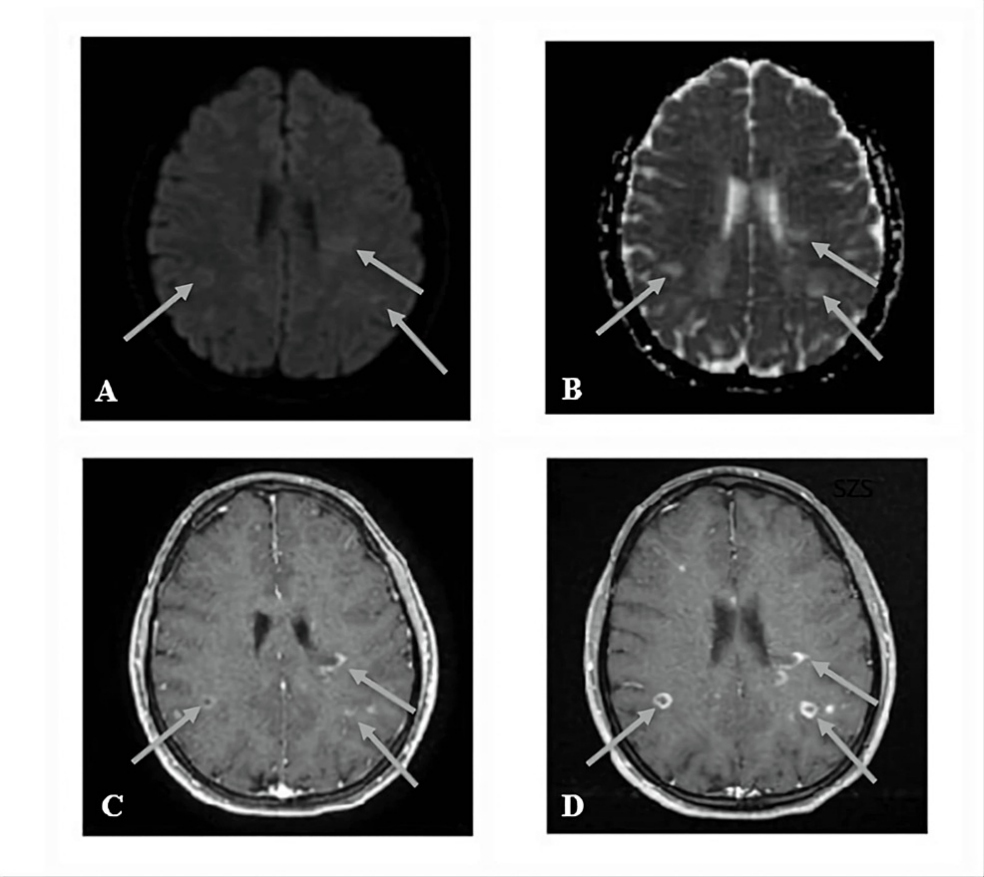
A 23-year-old female MS patient with multiple supratentorial brain parenchymal lesions (grey arrows). (A and B) Axial DWI and ADC map showed hyperintensity of the lesions. (C) Axial MPRAGE` with early enhancement showed faint enhancement. (D) Delayed enhancement of the lesions showing intense enhancement activity. MS, multiple sclerosis; DWI, diffusion-weighted image; ADC, apparent diffusion coefficient; MPRAGE, magnetization-prepared rapid acquisition gradient echo

For most of the active brain cases, there was a significant increase in the enhancement of the lesions with an increase in the signal intensity of DWI and the ADC map (Figure 5). Active MS brain lesions were significantly more enhanced in the delayed imaging time compared with the early enhancement time, with a P-value of <0.01 (Table 3 and Figures 6, 7).

**Table 3:** Early and delayed enhancement in the studied cases.

			Active (n=41)	Not active (n=59)	Total (n=100)	P-value
Enhancement time early in the brain	No	Count	35	59	94	<0.01
		%	85.4%	100.0%	94.0%	
	Yes	Count	6	0	6	
		%	14.6%	0.0%	6.0%	
Enhancement time delayed in the brain	No	Count	12	59	71	<0.01
		%	29.3%	100.0%	71.0%	
	Yes	Count	29	0	29	
		%	70.7%	0.0%	29.0%	

Spine findings

**Figure 8:**
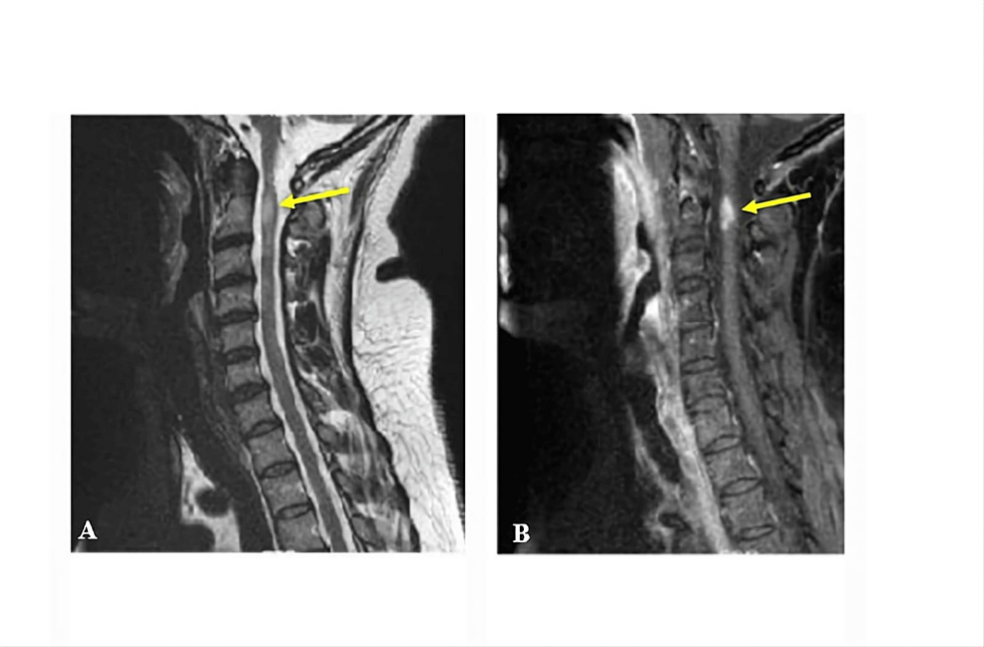
A 38-year-old female MS patient. (A) Sagittal T2-weighted imaging showing a bright signal intensity lesion in the cervicomedullary junction, left aspect of the cord opposite the C2 level. (B) T1-weighted imaging post-contrast administration demonstrating a intense enhancement lesion in the delayed phase.

**Figure 9:**
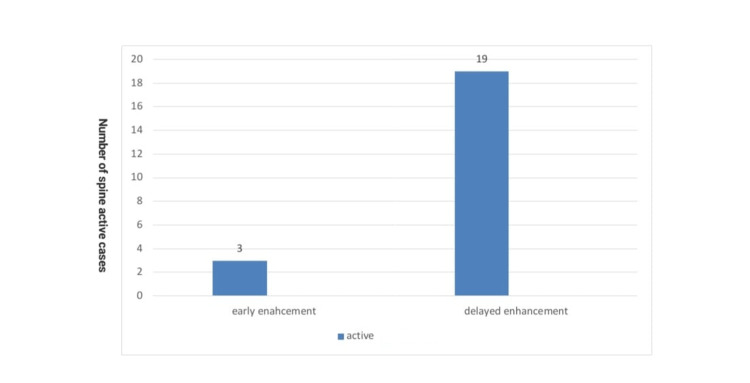
Early and delayed enhancement in spine in the studied cases.

Active MS spine lesions showed significantly high signal intensity in T2 images and significantly increased signal in post-contrast administration (Figure 8). Inactive spine cases did not show enhancement of the lesions after contrast administration. In the active spine lesions, there was a significant increase in the enhancement of the delayed image time compared to the early image time, with a P-value of <0.01. Active spine lesions showed more hyper-signal intensity in the pre-contrast T2 than in inactive cases, with a P-value of <0.01 (Table 4 and Figure 9).

**Table 4:** Early and delayed time in spine in the studied cases.

			Active (n=41)	Not active (n=59)	Total (n=100)	P-value
Enhancement time early in the spine	No	Count	38	59	97	0.066
		%	92.7%	100.0%	97.0%	
	Yes	Count	3	0	3	
		%	7.3%	0.0%	3.0%	
Enhancement time delayed in the spine	No	Count	22	59	81	<0.01
		%	53.7%	100.0%	81.0%	
	Yes	Count	19	0	19	
		%	46.3%	0.0%	19.0%	

## Discussion

This research shows that middle-aged females were more affected by MS than male patients, which was related to many different factors such as genetic and hormonal effects. For the genetic effect, the haplotype of HLA-DR2DQ6, which is associated with most autoimmune diseases and this disease, is more commonly found in women than men [46,47]. Unfortunately, genetic study of MS patients is not conducted routinely in our MS centers; thus, we do not have data about our participants.

For hormonal effects, there is a strong relationship between MS and female hormones [48,49]. Female hormones, such as estrogen, prolactin, progesterone, and androgens, which change during pregnancy, puerperium, puberty, and menopause impact the regulation of this disease and, as a result, have a significant impact on the frequency and development of the disease [50,51]. In men, it indicates a deficiency of testosterone, which increases the risk of this disease. Large levels of testosterone can prevent adolescents from developing this disease [52].

This study could prove how well CE images and DWI can distinguish between active and inactive MS lesions in the brain and spine. Based on previous research, the majority of earlier studies demonstrated the value of contrast enhancement time in the identification of active MS on MRI [53]. In addition, Granziera and Reich concluded that CE MRI is essential for determining disease activity, diagnosing MS, observing therapy, and evaluating the side effects of therapy. The regular use of GBCAs may prove less significant in some MS patients who show no signs of radiological or clinical disease activity [42].

Our results showed the significance of delayed enhancement time compared with the early time with a P-value of <0.01 in both the brain and spine. These results are similar to those reported by Pajouhan-Far et al., who assessed the reliability of MS diagnosis based on MRI results with IV contrast to improve knowledge of the variations in the delayed phase of MS [54]. They found that images acquired in the delayed phase in comparison to the early phase showed improved MS plaque detection. Similarly, these results were supported by Emara et al., who compared early and delayed T1 post-contrast images to evaluate the use of the delayed T1 post-contrast sequence in the detection of active lesions in MS patients. Higher results were found in the delayed time, in addition to increased MRI sensitivity, and demonstrated that delayed T1 post-contrast is a critical sequence for detecting active MS plaques [55].

The random Brownian motion of water molecules is described by the DWI sequence, which is controlled by a magnetic field gradient and reflects the signal density. The myelin that restricts the mobility of water molecules indicates tissue damage in MS. The higher ADC in DWI may indicate myelin breakdown because the pattern of water diffusion is altered because of the altered structural barrier [56]. Low ADC values are used for quantitative diffusivity; regions with low ADC and high DWI represent restricted diffusion [35].

In the current study, most of the active cases were enhanced in delayed time and showed a hyper-signal intensity in DWI, but the inactive cases were isointense in DWI. This is in agreement with the findings of Tsuchiya et al., who compared DWI with CE in MS patients. They showed that active lesions had an increase in enhancement after contrast administration with high signal intensity in DWI, while inactive lesions did not show enhancement and were iso- to hypo-intense [57]. However, this is not in agreement with the findings of Lo et al., who evaluated whether DWI may be used as an alternative to CE T1-WI for establishing DIT in MS. Their findings demonstrated that contrast enhancement did not always correspond with a hyperintense DWI finding. Several false positives could actually be other lesion growth phases. However, DWI might not be able to replace CE T1-WI [58].

Mohammed and Ismail noticed that chronic stable cases had low ADC values, and acute progressive cases had significantly higher ADC values [59]. This matches our findings regarding ADC map intensity for each of the active and inactive cases. In contrast, this is not in line with the findings of Unal et al., who determined the mean ADC values and signal intensity of DWI on active and inactive MS lesions. Both lesion groups had statistically significant increased ADC values, and there was no statistically significant difference between the active and inactive lesions [34].

Our research limitations were only related to MS of the spinal cord, including the non-application of DWI and post-contrast imaging at different intervals for every spine lesion, in addition to the low number of active MS of the spine. These limitations affected the statistical significance of MRI of the spinal cord in MS, which was mentioned in our recommendation.

## Conclusions

MRI is the gold standard non-invasive modality of choice for observing damage to the myelin sheath in MS patients. The use of CA has developed over the years in the diagnosis of MS patients. In the current study, active MS lesions show high signal intensity in both the DWI sequence and the CE delayed time, while inactive lesions appear to have low signal intensity in DWI with no enhancement after contrast injection whether early or delayed.

We recommend adding the following to the MRI protocol of MS patients: DWI sequence for suspected active MS spine lesions in addition to delayed enhancement time after contrast injection to increase MRI sensitivity toward active MS lesions of the brain and spinal cord as well.
